# Negative Facial Expressions – But Not Visual Scenes – Enhance Human Working Memory in Younger and Older Participants

**DOI:** 10.3389/fphar.2017.00668

**Published:** 2017-09-26

**Authors:** Flávia Schechtman Belham, Maria Clotilde H. Tavares, Corina Satler, Ana Garcia, Rosângela C. Rodrigues, Soraya L. de Sá Canabarro, Carlos Tomaz

**Affiliations:** ^1^Laboratory of Neurosciences and Behavior, Department of Physiological Sciences, University of Brasilia, Brasilia, Brazil; ^2^Institute of Cognitive Neuroscience, University College London, London, United Kingdom; ^3^Faculty of Ceilandia, University of Brasilia, Brasilia, Brazil; ^4^Euro-American University Center (UNIEURO), Brasilia, Brazil; ^5^Neuroscience Research Program, CEUMA University, São Luís, Brazil

**Keywords:** aging, visuospatial working memory, emotion, facial stimuli, IAPS

## Abstract

Many studies have investigated the influence of emotion on memory processes across the human lifespan. Some results have shown older adults (OA) performing better with positive stimuli, some with negative items, whereas some found no impact of emotional valence. Here we tested, in two independent studies, how younger adults (YA) and OA would perform in a visuospatial working memory (VSWM) task with positive, negative, and neutral images. The task consisted of identifying the new location of a stimulus in a crescent set of identical stimuli presented in different locations in a touch-screen monitor. In other words, participants should memorize the locations previously occupied to identify the new location. For each trial, the number of occupied locations increased until 8 or until a mistake was made. In study 1, 56 YA and 38 OA completed the task using images from the International Affective Picture System (IAPS). Results showed that, although YA outperformed OA, no effects of emotion were found. In study 2, 26 YA and 25 OA were tested using facial expressions as stimuli. Data from this study showed that negative faces facilitated performance and this effect did not differ between age groups. No differences were found between men and women. Taken together, our findings suggest that YA and OA’s VSWM can be influenced by the emotional valence of the information, though this effect was present only for facial stimuli. Presumably, this may have happened due to the social and biological importance of such stimuli, which are more effective in transmitting emotions than IAPS images. Critically, our results also indicate that the mixed findings in the literature about the influence of aging on the interactions between memory and emotion may be caused by the use of different stimuli and methods. This possibility should be kept in mind in future studies about memory and emotion across the lifespan.

## Introduction

Visuospatial working memory (VSWM), one of the storage subsystems of working memory (Baddeley and Hitch, 1974, 1994), has been found to be impacted by cognitive aging (Fabiani et al., 2015; Cabeza et al., 2016). Hale and colleagues, for instance, conducted a series of studies comparing age-related differences between visuospatial and verbal working memory (Jenkins et al., 2000; Myerson et al., 2003; Hale et al., 2011) and found stronger differences between younger (YA) and older adults (OA) in the visuospatial domain, suggesting that the latter is severely affected by aging (see also Bopp and Verhaeghen, 2007). Nevertheless, the effects of emotional processing in the age-related decline in VSWM have yet to be understood. In other words, although it is known that the modulatory effect of emotion on memory changes across the lifespan (Scheibe and Carstensen, 2010), it is not clear how these alterations occur in VSWM.

A common idea in the literature about how aging affects memory and emotion interactions is the Positivity Effect, which refers to how OA’s memory is biased toward positive events or stimuli (Carstensen and Mikels, 2005; Mikels et al., 2005; Petrican et al., 2008). A proposal by Mammarella et al. (2016a) offers a biological explanation for this effect, based on the modulatory influences of noradrenaline on emotional memory (Tully and Bolshakov, 2010). According to Mammarella et al. (2016a), recent evidence points to noradrenaline being linked to behavioral changes related to motivation, reward, and stimuli salience (Bouret and Richmond, 2015; Mather et al., 2015). Another line of evidence suggests an increase in activity of the noradrenergic system in aging (Seals and Esler, 2000), which would increase the effects of this component on OA’s emotional memory. Additionally, if aging brings a shift in a person’s goals, motivation, and interests, distinct stimuli may become more or less salient and rewarding. That is, if OA are more focused on emotionally meaningful and positive experiences, these will possibly be differently affected by noradrenaline compared to YA, leading to the Positivity Effect. In fact, such age-related change in goals, motivations, and interests is compatible with another proposal, named the Socioemotional Selectivity Theory (Carstensen et al., 1999). This theory states that one’s goals depend on their temporal context. Whereas YA perceive their remaining lifetime as long, OA tend to focus their cognitive resources on the pursuit of emotionally meaningful experiences. Joining Mammarella et al. (2016a)’s and Carstensen et al. (1999)’s proposals, OA’s focus on positive events may make these events more salient and differentially modulated by noradrenaline, enhancing their memorization.

Recent studies have supported the presence of a Positivity Bias in OA using different types of stimuli, including images from the International Affective Picture System (IAPS) (Lang et al., 1997;Mammarella et al., 2016b; Kan et al., 2017) and facial stimuli (Castel et al., 2016; Sava et al., 2017). Nevertheless, it is necessary to point out that some studies did not find a Positivity Effect. Those studies found a Negative Bias instead, with both YA and OA having better memory performances for negative items (Gruhn et al., 2005; Thomas and Hasher, 2006; Satler and Tomaz, 2011; Belham et al., 2013; Foster et al., 2013). This may happen because negative stimuli require prompter responses from an evolutionary point of view (Rozin and Royzman, 2001; Galli et al., 2011). Additionally, some studies simply have found no effect of emotion on memory whatsoever (Denburg et al., 2003; Garcia et al., 2011).

Regarding working memory specifically, recent studies have found mixed effects of emotion on OA’s performance. For example, Ziaei et al. (2015, 2017) found no effects of emotional valence when younger and older participants had to indicate if an IAPS image had been presented during the past three trials. Bermudez and Souza (2016), however, asked participants to indicate the six positions previously occupied by IAPS images in an array of 16 images. Valence did not affect YA’s behavior, but OA performed significantly worse with negative images. Mammarella et al. (2013) used a working memory task with emotional words and found that positive valence had a beneficial effect in OA but not in YA. Using the same task, Borella et al. (2014) found that both age groups performed better with negative words, although this result seemed to be influenced by individual differences. In a different study also using words, Truong and Yang (2014) found that negative and positive valence equally facilitated performance and the effects did not between the two age groups.

These mixed findings, together with the lack of knowledge about how OA’s VSWM is influenced by emotions, lead to the necessity of more studies. Here we aimed to investigate how YA and OA would perform in a VSWM test with negative, positive, and neutral stimuli. Younger and older participants responded the Spatial Delayed Recognition Span Task (SDRST) (Satler et al., 2015), in which identical stimuli are presented in different locations in a crescent set of up to eight locations. This task was chosen because it requires that participants keep the initial spatial locations in mind to be able to identify the new location within each trial. The locations are changed in each new trial and participants must update the information in their VSWM. This task has previously been used with YA and OA (Satler et al., 2015). We predicted that emotional valence would facilitate performance in both age groups. However, as detailed in the previous paragraph, the working memory literature shows mixed results in terms of how the age groups’ performance is modulated by the emotional valence of the stimuli. Thus, we did not have a strong prediction as to which valence (negative or positive) would have a larger influence in the current study. We report two independent studies using the same SDRST. Study 1 used negative, positive, and neutral images from the IAPS. In study 2, the stimuli were composed of angry, happy, and neutral facial expressions. We were interested in how accuracy in this VSWM task would be affected by the different valences displayed by the stimuli on the screen.

## Study 1

### Materials and Methods

#### Participants and Stimuli

Study 1 included 56 YA recruited from the university’s undergraduate programs (30 women; mean age 21.38 ± 2.90; at least 13 years of formal education) and 39 healthy OA (24 women; mean age 71.10 ± 6.72; at least 13 years of formal education) recruited from the Geriatric Medical Center, University Hospital of Brasilia. All were right-handed volunteers with no history of neurological or psychiatric episodes and no recent use of psychotropic medication, as evaluated by a detailed anamnesis. The eligibility criteria also included no consumption of alcohol or drugs in the 24 h prior to testing. Participants were vaguely informed about the aims of the study, and signed a written informed consent in accordance with the ethical guidelines for research with human subjects (196/96 CNS/MS Resolution). The study was approved by the Human Subjects Ethics Committee of the Health Sciences Faculty of the University of Brasilia (CEP-FS160/08 and CEP-FM064/2007). All participants had normal or corrected-to-normal vision and hearing. OA scored at least 24 on the Mini-mental State Examination (Folstein et al., 1975) and less than 9 on the Geriatric Depression Scale (Yesavage and Sheikh, 1986).

The stimuli material used in study 1 were composed of emotional pictures selected from the IAPS (Lang et al., 1997) and could depict objects and scenes. Three positive images (slides number: 5750, 5030, 1660; valence: 6.60, 6.51, 6.49; and arousal: 3.14, 2.74, 4.57, respectively), four negative images (slides number: 3000, 3120, 3130, 3030; valence: 1.41, 1.56, 1.58, 1.91; and arousal: 7.26, 6.84, 6.97, 6.76, respectively), and two neutral images (slides number: 5510, 7010; valence: 5.15, 4.94; and arousal: 2.82, 1.76, respectively) were selected. No facial images were selected for study 1. Each participant responded to nine trials, one for each image, presented in a pseudo-randomized order. Computer software registered correct and incorrect responses for each given answer. The time of execution of the task varied according to each participant’s response time, but the full procedure did not last more than 2 h.

#### The Spatial Delayed Recognition Span Task (SDRST)

The task was a computer-based version (Delphi language, computational program TREA) of the SDRST, which measures participants’ working memory (Lacreuse et al., 2005). The task is presented to YA and OA on a touch-screen monitor (LG Studio Works 440, Microtouch, 17′) positioned within arm’s reach. The computer-based SDRST has been successfully used with different populations and stimuli by our group (Satler et al., 2015). In this task, participants must discriminate a novel location of a stimulus among an increasing array of identical stimuli presented sequentially in various locations within the same trial. At the beginning of each trial, one stimulus is presented at random in 1 of the 16 possible locations on the screen. Participants must touch it. After a pre-determined delay, that stimulus re-appears in the same position and another identical stimulus appears in a new position. Participants must touch the stimulus presented in the new location. Every time a correct response is made, a new stimulus is added to the array. This goes on until the maximum of eight stimuli or until a mistake is made. In both cases, a new trial with a different stimulus begins. Correct answers led to the emission of an acute auditory feedback signal; wrong answers led to a bass auditory signal. Stimuli within a single trial were identical and did not repeat in two consecutive trials.

**FIGURE 1:**
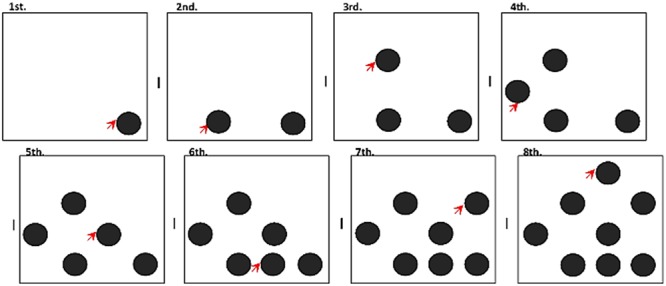
Experimental design for the Spatial Delayed Recognition Span Task (SDRST). Participants had to memorize the occupied locations and identify the new location within each trial up to a maximum of eight locations. The task was conducted on a touch-screen monitor.

The stimuli were presented for a period of up to 5 s (1 s for YA, 5 s for OA) (**Figure 1**). Before the beginning of the task, every participant received written and oral instructions and completed a practice session. The practice session used geometrical shapes as stimuli and was conducted in the same fashion as the main task. The practice session was concluded when participants correctly answered two consecutive complete trials (eight stimuli per trial) or after 20 trials.

#### Statistical Analyses

Accuracy was calculated as the mean of correct choices before a mistake for all the trials of each emotional valence. A mixed-design ANOVA was run (SPSS v. 18.00; SPSS, Inc., Chicago, IL, United States, 2009) with age (YA or OA) as a between-subjects factor, and emotional valence (neutral, positive, and negative) as a within-subjects factor. Significance was defined as a *p*-value < 0.05.

### Results

**FIGURE 2:**
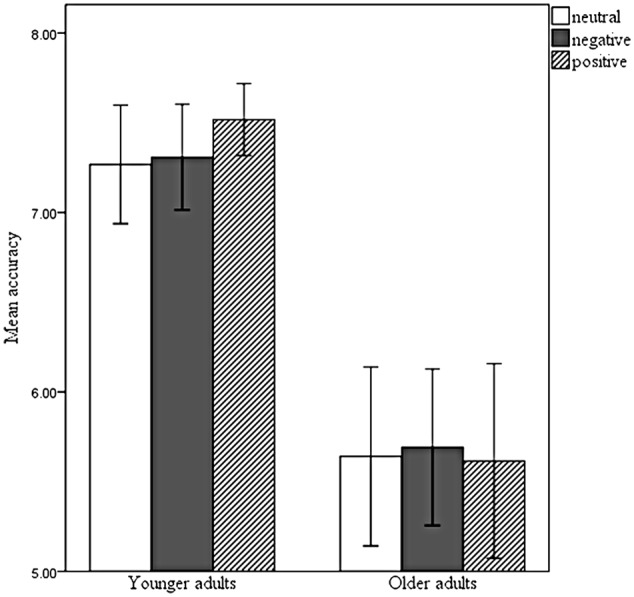
Mean accuracy for younger (YA) and older adults (OA) responding to the SDRST with neutral, negative, and positive IAPS images. Error bars represent confidence intervals (95%). YA outperformed OA (*p* < 0.001). There was no significant influence of emotional valence (*p* = 0.706).

Mean accuracy for YA and OA can be seen in **Table 1**. No differences were found between men and women (*p* > 0.205). Accuracy in this task was not influenced by the emotional valence of the IAPS image (*F*_(2,186)_ = 0.293, *p* = 0.706, $\eta_{p}^{2}$ = 0.004). However, YA outperformed OA (*F*_(1,93)_ = 65.217, *p* < 0.001, $\eta_{p}^{2}$ = 0.412). There was no significant interaction between the factors (*F*_(2,186)_ = 0.723, *p* = 0.487, $\eta_{p}^{2}$ = 0.008) (**Figure 2**).

**Table 1:** Accuracy during the Spatial Delayed Recognition Span Task (SDRST) for Younger adults and Older adults with positive, neutral, and negative IAPS images.

	Positive	Neutral	Negative	Average
Younger adults (*n* = 56)	7.52 (0.75)	7.27 (1.23)	7.31 (1.10)	7.36 (0.77)
Older adults (*n* = 39)	5.61 (1.67)	5.64 (1.54)	5.69 (1.34)	5.65 (1.29)
Average	6.74 (1.53)	6.60 (1.58)	6.64 (1.44)	

*Mean (SD) of a maximum of 8.*

### Discussion

The age-related difference in performance found in study 1 is in line with previous research, suggesting that healthy aging is related to deficits in various cognitive domains, including VSWM. For instance, Jenkins et al. (2000) tested YA and OA in visuospatial speed, memory, and learning tasks and discovered an age-related decline on all three types of processing.

The absence of a valence effect on memory performance was surprising and unexpected. As mentioned before, several studies support the existence of a Negativity Bias in YA and a Positivity Effect in OA. Nevertheless, the current results are in line with other investigations reporting no valence effects on memory performance, such as Denburg et al. (2003) and Garcia et al. (2011). The present results are also in line with at least other two studies who found no effects of emotion on YA and OA’s WM for IAPS images (Ziaei et al., 2015, 2017).

One possibility is that the non-significant effect of valence in the current study was caused by the fact that the IAPS images are not a straightforward way of displaying emotions, being sometimes too complex or rich in visual information (Britton et al., 2008). By contrast, facial photographs with different emotional expressions are considered by many studies as one of the most important and direct ways of externalizing emotions (Hess et al., 1997; Nahm et al., 1997). For example, Isaacowitz et al. (2007) investigated age-related differences in the recognition of emotions from lexical stimuli (sentences describing emotional situations) and facial expressions. They found an interaction between age group and task type, indicating that facial stimuli elicited significantly less age differences than did the lexical stimuli. Some researchers, on the other hand, propose that aging brings deficits in the recognition of negative facial expressions, but not of positive ones (Orgeta and Phillips, 2007; Kellough and Knight, 2012). Moreover, Altamura et al. (2016) found that OA are quicker in identifying a positive facial expression compared to a negative one. Thus, we decided to conduct a second study using photographs of facial expressions as stimuli. Due to the social and biological relevance of facial stimuli, we predicted that study 2 would lead to different results from study 1, with emotional valence influencing memory performance of the two age groups.

## Study 2

### Materials and Methods

Inclusion and exclusion criteria were the same as used in Study 1. Twenty-six YA recruited from the university’s undergraduate programs (13 women; mean age 21.31 ± 2.05 years; at least 14 years of formal education) and 25 healthy OA (11 women; mean age 69.92 ± 6.41 years; at least 13 years of formal education) recruited from the Geriatric Medical Center, University Hospital of Brasilia took part in this study. This study was approved by the Human Subjects Ethics Committee of the Health Sciences Faculty of the University of Brasilia (CEP-FS 097/11). For this study, all participants scored more than 24 on the Mini-mental State Examination (Folstein et al., 1975) and less than 9 on the Geriatric Depression Scale (Yesavage and Sheikh, 1986).

The same SDRST was conducted (**Figure 1**) using colored photographs (4 cm × 4 cm) of adult models displaying different facial expressions, manipulated to only show the face, with no interference from hair or other body parts. The facial data set was provided to our group by Dr. Hisao Nishijo from the University of Toyama, Japan, and has been used in previous studies from that lab (Hori et al., 2005). Seven images of happiness (positive valence), two images of anger (negative valence), and two neutral expressions were chosen from the original data set based on a pilot study where 30 YA and OA (who did not participate in the main study) identified the emotional expression of several faces displayed on the screen. Only images that elicited a correct classification rate of over 90% were chosen. Each participant responded to one block of each emotional valence. Each block contained 10 trials and two identical faces were never presented in two consecutive trials. Images were presented for 3 s.

### Results

**FIGURE 3:**
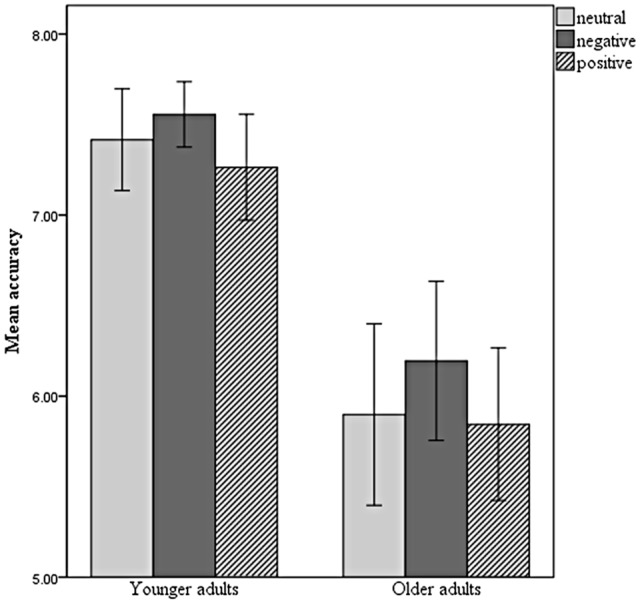
Mean accuracy for YA and OA responding to the SDRST with neutral, negative, and positive facial photographs. Error bars represent confidence intervals (95%). YA outperformed OA (*p* < 0.001). Performance with negative images was higher than with positive images (*p* = 0.002).

Mean accuracy for YA and OA can be seen in **Table 2**. No differences were found between men and women (*p* > 0.107). Accuracy was influenced by the valence of the facial expression (*F*_(2,98)_ = 6.024, *p* = 0.003, $\eta_{p}^{2}$ = 0.109). Pairwise comparisons adjusted with the Bonferroni correction showed that negative items elicited much better performance than positive items (*p* = 0.002) and a marginally better performance than neutral items (*p* = 0.084). Neutral items did not differ from positive items (*p* = 0.894). YA performed better than OA (*F*_(1,49)_ = 40.198, *p* < 0.001, $\eta_{p}^{2}$ = 0.451). There was no significant interaction between the factors (*F*_(2,98)_ = 0.351, *p* = 0.705, $\eta_{p}^{2}$ = 0.007) (**Figure 3**).

**Table 2:** Accuracy during the SDRST for Younger adults and Older adults with positive, neutral, and negative facial stimuli.

	Positive	Neutral	Negative	Average
Younger adults (*n* = 26)	7.26 (0.72)	7.42 (0.70)	7.56 (0.45)	7.41 (0.54)
Older adults (*n* = 25)	5.84 (1.02)	5.90 (1.22)	6.19 (1.06)	5.98 (1.01)
Average	6.57 (1.13)	6.67 (1.24)	6.89 (1.06)	

*Mean (SD) of a maximum of 8.*

### Discussion

In study 2, YA again performed better than OA, as expected. However, this time valence influenced memory accuracy, with negative faces eliciting a better performance than positive faces and neutral faces (marginally). This is called the Negativity Bias. It states that, due to their larger influence on the adaptive value of an individual, negative events are more efficiently remembered (Rozin and Royzman, 2001). Negative stimuli also attract more attention to their location and generate a more prompt behavioral response because they indicate places to be avoided, possibility of contamination, and imminent threats from others (reviewed by Palermo and Rhodes, 2007). Being in a negative emotional state has also been shown to improve cognition (Gray et al., 2002). Importantly, study 2 revealed no interaction between age and emotion, suggesting that negative emotion can benefit memory performance of both YA and OA, as found in previous studies (Gruhn et al., 2005; Thomas and Hasher, 2006; Foster et al., 2013). In other words, the results of the current study suggest that YA and OA did not differ in how their VSWM is influenced by the emotional valence displayed in facial stimuli.

The absence of the Positivity Effect in OA may have been due to the difficulty of the task. Two studies (Mather and Knight, 2005; Knight et al., 2007) presented participants with either full-attention tasks or divided-attention tasks and showed that, when the task is more cognitively demanding, the tendency of OA to favor positive over negative images is eliminated. It is also possible that the Positivity Effect is not as universal as originally thought, since the current study and others studies mentioned throughout this paper have failed to find it.

On a different note, Lewinski (2015) argues that humans only rate neutral facial expressions as neutral in 60% of the cases. This result does not suggest that the neutral faces used in the current study were inappropriate. Rather, it raises the possibility that the 30 pilot participants failed to recognize other faces as neutral. Future research may take advantage of automated facial coding software to aid in stimuli selection.

## General Discussion: Iaps × Faces

The present research explored the responses of YA and OA performing a VSWM task that required the processing of emotional stimuli. The main goal was to investigate age-related differences in the emotional modulation of VSWM. Study 1 used positive, negative, and neutral IAPS pictures, whereas study 2 presented participants with happy, angry, and neutral facial expressions. As expected, in both studies YA showed higher accuracy than OA. Interestingly, however, only in study 2 emotion influenced memory, with negative faces leading to a better performance. The results were not influenced by whether participants were male or female, suggesting no sex-related differences in the relationship between VSWM and emotion.

A few possible explanations for the different results between the two studies can be raised, based on how faces differ from other visual stimuli. First, faces are one of the most important and straightforward ways of externalizing emotions (Hess et al., 1997). When directly compared with IAPS images, faces are less ambiguous and more familiar, which enhances the efficiency of their processing (Öhman et al., 2001; Britton et al., 2008; Ekman and Cordaro, 2011). Second, studies have demonstrated that faces are more quickly detected than other types of stimuli (Palermo and Rhodes, 2007), partially due to the Fusiform Face Area (Kanwisher et al., 1997). Liu et al. (2002) showed that a stimulus is categorized as a face in extricate areas of the brain no longer than 100 ms after the stimulus presentation, which does not happen for other images (e.g., houses). The fact that facial stimuli are more quickly identified and do not require additional cognitive load (Britton et al., 2008) may have allowed for a faster processing of the emotional information in those stimuli and, in consequence, strengthened their influence on VSWM when compared to IAPS images. Finally, IAPS images are more complex and more distinct between each other than faces. These characteristics induce a slower habituation to novelty and demand more sustained attention during the task (Knight et al., 2007; Britton et al., 2008). Previous research has shown that the effects of emotion on memory are reduced when participants are instructed to pay extra attention to stimuli (Talmi et al., 2008). Thus, the additional attentional resources required by the IAPS images may have reduced the influence of their emotional content on memory, when compared to emotional faces. All this evidence supports the conclusion that faces are more efficient in transmitting emotional valence and, thus, influencing working memory, than other types of stimuli such as contextual pictures.

Some limitations of the current study should be addressed. Our experiments were not designed to directly test the influence of the type of stimuli on memory and emotion interaction in OA. Study 1 was designed to investigate VSWM for emotional images and study 2 was developed in consequence of the results from the first study. Also, in study 1, the stimuli presentation times differed between the two age groups due to OA being slower in their movements and less familiar with the use of the computer. In study 2 we used the same stimulus duration for both age groups. Because of this, a direct comparison between IAPS and facial stimuli in the same study should be conducted to strengthen our conclusions. The facial stimuli used in our studies showed pictures of adult models, but not of OA. This means that our older participants were responding to out-group faces, which could lead to different cognitive processing (Samanez-Larkin and Carstensen, 2011). However, previous research has demonstrated that both YA and OA are faster and more accurate in identifying emotional expressions in younger than in older faces (Ebner et al., 2012). This finding, and the fact that we found no interactions between emotion and aging, supports the conclusion that the facial stimuli used here were adequate, though future research could directly repeat our experiments using older faces. It is important to highlight that the use of only anger as the negative emotional expression was dictated by the pilot study described in the “Materials and Methods” section of study 2. However, we are aware that the processing of negative valence may differ due to the specific emotion being tested (Phan et al., 2002). Thus, it is important that future studies repeat our experiments with a larger variety of facial expressions (e.g., sadness and disgust) within the same emotional valence.

## Conclusion

Our findings suggest that the emotional modulation of VSWM is influenced by the type of information to be remembered. This effect seems to be present for YA and for OA. Taken together, our results contribute to the understanding of information processing in YA and OA and to the characterization of cognition across the human lifespan. This knowledge may explain the inconsistent literature findings about the interplay between memory, emotion, and aging, and lead to the development of better methodological approaches when studying these topics.

## Author Contributions

FB, MT, CS, AG, and CT: designed study. FB, CS, and AG: collected data. FB, MT, CS, AG, and CT: analyzed data. FB, MT, CS, AG, RR, SC, and CT: interpreted data. FB, MT, CS, AG, RR, SC, and CT: wrote paper.

## Conflict of Interest Statement

The authors declare that the research was conducted in the absence of any commercial or financial relationships that could be construed as a potential conflict of interest.
